# An Active System for Visually-Guided Reaching in 3D across Binocular Fixations

**DOI:** 10.1155/2014/179391

**Published:** 2014-02-04

**Authors:** Ester Martinez-Martin, Angel P. del Pobil, Manuela Chessa, Fabio Solari, Silvio P. Sabatini

**Affiliations:** ^1^Robotic Intelligence Lab, Department of Engineering and Computer Science, Universitat Jaume-I, 12071 Castellón, Spain; ^2^Interaction Science Department, Sungkyunkwan University, Seoul 110-745, Republic of Korea; ^3^Department of Informatics, Bioengineering, Robotics and System Engineering (DIBRIS), University of Genoa, 16145 Genoa, Italy

## Abstract

Based on the importance of relative disparity between objects for accurate hand-eye coordination, this paper presents a biological approach inspired by the cortical neural architecture. So, the motor information is coded in egocentric coordinates obtained from the allocentric representation of the space (in terms of disparity) generated from the egocentric representation of the visual information (image coordinates). In that way, the different aspects of the visuomotor coordination are integrated: an active vision system, composed of two vergent cameras; a module for the 2D binocular disparity estimation based on a local estimation of phase differences performed through a bank of Gabor filters; and a robotic actuator to perform the corresponding tasks (visually-guided reaching). The approach's performance is evaluated through experiments on both simulated and real data.

## 1. Introduction

A long-term goal of Robotics research is that of building robots which behave and even look like human beings. So, aimed at working with and for people, human abilities should be modelled and replicated in a robotic system. In that way, robots should be able to complete their tasks by properly interacting with their environment [1]. As in the case of human beings, those interactions in space should be *explicit*, (e.g., pointing, reaching, or grasping things) as well as *implicit* (in the sense of achieving an awareness of *where* and *what* things are around them).

In this regard, visual information has been extensively used to control a robot system by increasing its flexibility and accuracy (e.g., [2–10]). However, this approach, commonly known as *visual servoing*, keeps separate the vision and motion control processes, so that image processing *simply* provides the *error* signals required by the actual control schemes. As a matter of fact, all these techniques are based on separate or mildly interacting modules. In addition, a key restriction of this approach is the image processing of natural views, that is, the extraction of robust features for visual servoing. On the contrary, the concept beyond this paper is to investigate if visual processing and ocular movements, as well as more general robot motions, could be integrated at different levels to improve the interaction capability in the robot *peripersonal space* by properly modelling the observed scene.

As a solution, we have taken advantage of the concept of active vision [11, 12] since it is exploratory and predictive. Actually, in that way, a robot can evolve from a status of passive observer overawed by information to a more selective agent able to control and adapt its own perception according to the task to be performed. As an example, Coombs and Brown [13] demonstrated how dynamic vergence control could cleverly interact with image processing for tracking moving targets over cluttered backgrounds. Note that the vergence movements adjust the eyes for viewing objects at varying depth. So, while the recovery of absolute depth cannot be strictly necessary, the relative disparity between objects is critical for tasks such as accurate hand-eye coordination, figure-ground discrimination, and/or collision detection. Furthermore, disparity provides some cues for planning and controlling goal-directed behaviours.

So, our research is aimed at exploiting the interaction existing between vision and motion control to achieve a knowledge of the surrounding space when reaching a visual object is the task. For that, it is necessary to design and implement a space representational scheme that supports a natural behaviour flexible enough to deal with how the robot's actions influence the world. In other words, this paper presents a biological strategy endowing a robotic system with basic visuomotor behaviours, perceptual abilities, and manipulative abilities (i.e., reaching for a visual target). Therefore, the designed robotic system could robustly perform visuo-motor tasks in complex, natural environments without any *a priori* knowledge.

### 1.1. The Biology of Spatial Coding

From a biological point of view, the interaction strategy apparently adopted by all the superior vertebrates consists of separating the recognition of an object (the *what* problem) from finding its position (the *where* problem). So, the temporal regions of the cerebral cortex are involved in the *what* pathway, while the parietal regions try to find *where* the interest objects are [14–16]. The parietal system can be then regarded as an acting strategy to focus the system's attention on a particular zone of the perceptive field. This approach leads to a *from-action-to-perception* scheme [17–19]. That is, action and perception are linked such that actions can modify perceptions *externally* and *internally*. In other words, performing an action externally influences the perception by changing the scene and/or the point of view (e.g., the movement of the eyes serves to choose a scene for perception). At the same time, this can imply an internal modification of the perception since different information can be required to properly plan and execute the next action. As a consequence, percepts and actions can be coupled at different levels such that the proper combination of them provides a complete and operative cognition of the surrounding space [20, 21].

**Figure 1:**
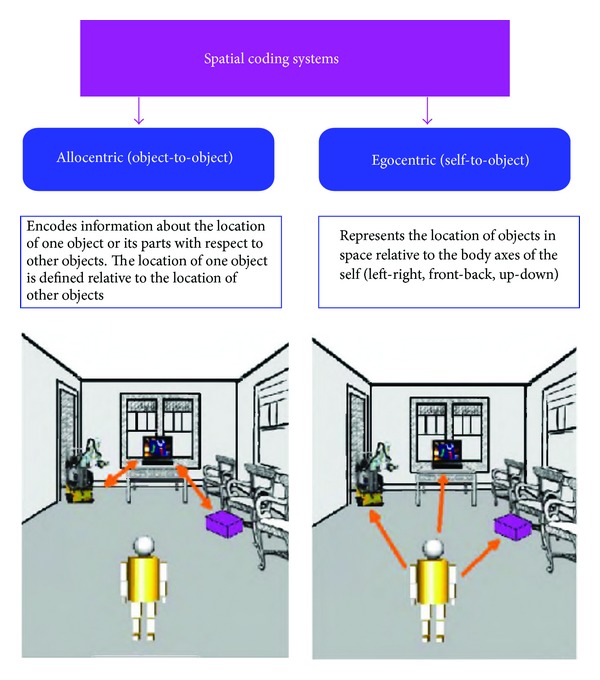
Allocentric versus egocentric spatial processing. Allocentric spatial transformations involve an object-to-object representational system and encode information about the location of one object or its parts with respect to other objects, while egocentric perspective transformations involve a self-object representational system.

In this context, the key question is *how does the brain achieve perceptual stability despite the nature of the input supplied by the eyes?* Actually, this question has been asked by researchers since the *saccade-and-fixate* strategy of the oculomotor system was first observed [23]. Recent accounts of the way humans encode information about objects, places, and routes in the world around them propose that they have two kinds of spatial representation: *allocentric* and *egocentric* [24–26] (see Figure 1). As defined in [22], the *allocentric* representation is map-like. It is indexed to a world-based coordinate system and, therefore, it is independent of a person's current location and it survives over extended periods of time. This representation must be built up from vision over time, but does not rely on immediate visual input. The other kind of spatial representation, that is, the *egocentric* representation, is temporary, and it is based on the object directions relative to the current body's position with respect to the surrounding space. This second representational frame allows humans to act upon their environment for the purposes of locating, reaching, and/or manipulating objects.

This egocentric-allocentric division follows a well-established neuropsychological distinction between the dorsal and ventral visual processing streams [20, 27]. Actually, these two frames of reference have specific functions in the vision-for-action and vision-for-perception model such that egocentric representations would be used by the dorsal stream to program and control the skilled movements needed to carry out the action, whereas conscious perception would rely on allocentric representations supported by the ventral stream [28, 29]. However, a new question arises: how they interact and combine [30].

**Figure 2:**
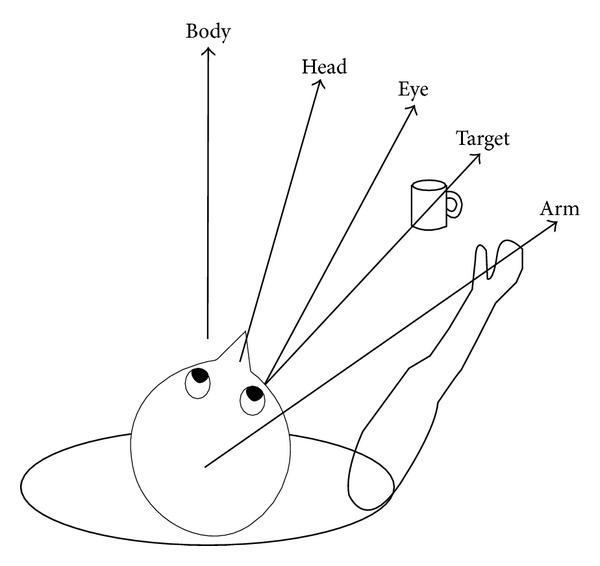
Frames of reference for visuomotor tasks. The required movement to grasp the mug is the angle from arm to target. This is the angle from body-to-arm minus the sum of the angles from target-to-fovea, eye-in-head, and head-on-body. In practice, eye, head, and body are often aligned before such a grasp movement, but such alignment is not essential (courtesy of Tatler and Land [22]).

Research on this topic [22, 24, 31–36] establishes that mental processes form a hierarchy of mental representations with maximally egocentric representations at the bottom and maximally allocentric representations at the top, progressively abstracting away from the particularities of the egocentric representations. So, visual information must be initially coded in retinotopic space, while muscular movement plans must be ultimately coded in head-centred and/or body-centred representations. Indeed, it is clear that, in the context of natural behaviour, a range of different spatial coding schemes are involved and they act in parallel (see Figure 2). This is the case, for instance, of arm reaching plans, which are encoded in eye-centred coordinates [37, 38]. However, it seems likely that efficient coordination of sensory input and motor output involves a transformation between the various parallel reference frames for spatial coding through the parietal cortex.

### 1.2. Contributions

In this paper, we propose a biological approach following the neural architecture such that the motor information to perform the task in hand is coded in egocentric coordinates (motor coordinates) obtained from the allocentric representation of the space (in terms of disparity) generated from the egocentric representation of the visual information (image coordinates). With that purpose, an active vision paradigm is used: the behaviour-dependent processing of visual data for attentive visual scrutiny based on shifting the fixation point of different targets (active foveation). So, the different aspects of the visuo-motor coordination are integrated: an active vision system, composed of two vergent cameras, a module for the estimation of 2D binocular disparity, and a robotic actuator to perform reaching tasks. Thus, the main contribution of this paper can be summarized in two points.

- Design and implementation of an algorithm (PBBDE) for disparity estimation that does not require precise calibration information (in terms of the relative orientation of the cameras).
- Design and implementation of a virtual reality tool to evaluate the performance of this method and to study the adaptation of robots behaviour in reaching tasks triggered by 3D perception in an unstructured environment.

These goals have been achieved by carrying out the following.

- A design and implementation of an architecture inspired by the cortical neural architecture aimed at a *more natural* robot interaction with the environment.
- An integration of different aspects of the visuo-motor coordination: an active vision system, a module for 2D binocular disparity and depth estimation, and a robotic actuator to perform reaching tasks.
- Robustly performing visuo-motor tasks in complex, natural environments without any *a priori* knowledge.
- A design and implementation of robotic perceptual and manipulative abilities (i.e., reaching for a visual target) by integrating visual processing, ocular movements, and robot motions at different levels without separating vision and motion control processes as in visual servoing.
- A design and implementation of visual targets depth.
- A design and implementation of a virtual reality tool that allows us to study robot behaviour adaptation on reaching task from 3D perception in an unstructured environment.
- An analysis of parameters that make the disparity map conditional to accuracy.
- A study of the computational cost of the proposed approach based on image size.

With that aim, this paper is organized as follows. In Section 2 we introduce the phase-based approach used for stereo processing in its generalized form to compute 2D disparity for vergent vision systems. A virtual reality tool implementing robotic reaching tasks from stereo visual cues is described in Section 3, while the experimental results, under different conditions, are presented in Section 4 and discussed in Section 5.

## 2. Stereo Processing

As mentioned above, disparity is an important cue for depth estimation since it provides an allocentric spatial representation allowing us to determine *absolute* distances when camera orientations are known.

Focusing on obtaining a *disparity map*, the first issue to be solved is the correspondence problem. Basically, it refers to the problem of matching *corresponding* image points in a stereo pair of images. Despite the large number of proposed algorithms (see [39–42] for an overview), they can be classified into two main groups, as pointed out in [40].

- *Area-based* matching algorithms. Image domain similarity metrics are used for dense point to point correspondence. Therefore, the resulting disparity map can be very dense what makes this kind of methods an interesting way to quantify and solve early vision problems.
- *Feature-based* matching algorithms. They concern the two following steps.
  - *Feature extraction.* Features such as colour, edges, and so forth are extracted from the images. The localization of these features is important, since disparities will be determined according to differences in position after the following step (i.e., the correspondence problem) has been solved.
  - *Solving the correspondence problem.* A correspondence between image elements is chosen from the many conceivable ones. Various types of knowledge, constraints, and plausibility considerations are used at this stage such as
    - search space: for an element in the left image, a matching element is sought only within a certain region of the right image,
    - feature attributes: in the case of the image elements can be distinguished from one another, then only those of the same type (e.g., edges, line terminations) and with the same characteristics (e.g., colour, polarity of contrast) are matched,
    - ordering constraints: the plausibility of other matches changes once a match between two features has been established. Consequently, constraints must be reorganized to extract depth information.

Note that this method results in sparse disparity maps since it only gets disparities for the extracted features

However, matching-correspondence methods usually cannot be efficiently adapted to changing camera's geometry information. For that reason, nearly all the proposed stereo vision algorithms separate the calibration and dense disparity estimation stages. On the one hand, regarding the calibration step, it is typically performed offline by means of feature-based techniques. Note that the calibration information is used for stereo rectification resulting in a simplified, faster matching process (from two dimensions to one). On the other hand, estimating the epipolar geometry from noisy correspondences, possibly including many outliers, is problematic. As an improvement of the calibration accuracy, either a special calibration object is used or the information of multiple image pairs is combined as in [43, 44]. Moreover, epipolar geometry estimation is usually stabilized by exploiting physical restrictions on the camera configuration. Thus, for instance, Björkman and Eklundh [45] presented a system to externally calibrate a stereo pair by assuming fixation and no rotations around the line of sight. On the contrary, Papadimitriou and Dennis [46] proposed a self-rectification method that focuses only on the removal of the vertical displacements. They assume a convergent camera system where only rotations around an axis parallel to the vertical axis (pan) need to be compensated. That reduces the problem and stabilizes the camera geometry estimation. However, as Papadimitriou and Dennis stated [46], vertical disparity can cause serious errors in matching process if the stereo images are not rectified very well. Therefore, a robust rectification must be used to obtain an accurate image matching correspondence, which is performed after the calibration stage. As an example, Gao et al. [47] proposed a real-time embedded system combining disparity estimation and self-rectification. As in [46], the system only corrects vertical shifts.

On the other hand, biological studies have revealed that the response of visual cortex is turned to the band-limited portion of the frequency domain. This fact gives evidence that the brain decomposes the spectra into perceptual channels that are bands in spatial frequency [48]. So, images can be seen as sinusoidal functions moved in depth and disparity can be extracted by means of frequency filters. In this context, Gabor functions have been extensively used due to their similarity with the receptive field of cells in the virtual cortex [49, 50]. Actually, they have been particularly successful in many computer vision and image processing applications [51–55]. However, a fundamental problem with these methods is the inherently large memory and computational overheads required for training and testing in the over-complete Gabor domain.

As an alternative, different band-pass filters based on specific properties of the basis functions [56–62], or according to theoretical and practical considerations of the whole space-frequency transform [63–72], have been proposed. Nevertheless, these techniques are very time consuming and hardly suitable for real-time applications. Furthermore, with Cartesian images, if the object of interest is small, the background disparity can lead to erroneous estimates. Alternatively, with space variant images, the target region becomes dominant [73].

Consequently, in this paper, we present an algorithm for *disparity* estimation that does not require precise calibration information (in terms of the relative transformation (position and orientation) between the two cameras). That is, the proposed approach does not use the external camera parameters. Consequently, cameras are only calibrated at the beginning of the experiment to obtain internal camera parameters, and no more calibration procedure is performed although the cameras shift their fixation point. For that, an active vision paradigm is used: the behaviour-dependent processing of visual data based on shifting the fixation point of different targets (active foveation) for attentive visual scrutiny. Selective attention and foveation imply the ability to control the mechanical and optical degrees of freedom during image acquisition process [74]. In such systems, the camera movements bring the object of interest in the centre of the image pair (by performing camera rotations), and these vergence movements generate both horizontal and vertical disparity [75–77].

### 2.1. Phase-Based Binocular Disparity Estimation (PBBDE) Approach

The difference in target's position in the two stereo images defines a *disparity* shift. That difference can be used to shift the left (or right) image to align both of them at the same coordinate location.

Assuming that an image is a sinusoidal gray value function moved in depth, the same gray value function appears in both images of a stereo pair at different phase angles. So, if the wavelength of the sinusoidal pattern is known, the phase difference corresponds to the *disparity*. Actually, this kind of approache can be used with any gray value functions, by filtering out all but one frequency band from the image [65, 78–85]. It has been shown that phase-based methods are robust to changes in contrast, scale and orientation [78]. The robustness to orientation is very important in the context of disparity estimation since textures or features on slanted surfaces have a different orientation in the left and right images.

To obtain the corresponding phase difference at a point *x*, a symmetrical and an antisymmetrical filter kernel are used, performing local estimations of the phase difference. So, for instance, the two filter outputs for the left image *I*_*l*_ would be

(1) $$\begin{array}{c} I_{l,\sin,\sigma }\left(x,w\right)=\int_{}^{}w\left(\frac{x-x^{\prime }}{\sigma }\right)I_{l}\left(x^{\prime }\right)\sin\left(w\left(x-x^{\prime }\right)\right)dx^{\prime },\\ I_{l,\cos,\sigma }\left(x,w\right)=\int_{}^{}w\left(\frac{x-x^{\prime }}{\sigma }\right)I_{l}\left(x^{\prime }\right)\cos\left(w\left(x-x^{\prime }\right)\right)dx^{\prime }, \end{array}$$

where *w* refers to the frequency of the kernel filter and *σ* corresponds to its spatial expansion. If the window function is the Gaussian bell curve and the ratio between *ω* and *σ* is a constant, then (1) describes a convolution with Gabor functions. In particular, the proposed method extracts phase using a bank of oriented Gabor filters by using a coarse-to-fine approach. Note that the proposed method takes into account the *x*-*y* image dimensionality by using a bank of two-dimensional oriented filters. In this way, an accuracy improvement has been obtained, as will be shown in Section 4.1.

The different orientations, *θ*_*q*_, are evenly distributed and equal to (*qπ*)/*K*. Let *q* be the range from 0 to *K* − 1, while a total of *K* = 8 orientations are considered in our implementation. Thus, for a specific orientation *θ*_*q*_, the spatial phase at pixel location **x** = (*x*,*y*)^*T*^ is extracted using 2D complex Gabor filters:

(2) $$\begin{array}{c} f_{q}\left(x\right)=e^{-(x^{2}+y^{2})/2\sigma^{2}\;}e^{j\omega_{0}(x\cos\theta_{q}+y\sin\theta_{q})} \end{array}$$

with peak frequency *ω*_0_ and spatial extension *σ*. The filter bank has been designed with efficiency in mind and relies on 11 × 11 separable spatial filter kernels that are applied to an image pyramid [83, 86]. The filter responses, obtained by convolving the image, *I*(**x**), with the oriented filter from (2), can be written as

(3) $$\begin{array}{c} Q_{q}\left(x\right)=\left(I*f_{q}\right)\left(x\right)=\rho_{q}\left(x\right)e^{j\phi_{q}(x)}=C_{q}\left(x\right)+jS_{q}\left(x\right), \end{array}$$

where $\rho_{q}\left(x\right)=\sqrt{C_{q}{\left(x\right)}^{2}+S_{q}{\left(x\right)}^{2}}$ and *ϕ*_*q*_(**x**) = arctan(*S*_*q*_(**x**), *C*_*q*_(**x**)) are the amplitude and the phase components, respectively, and *C*_*q*_(**x**) and *S*_*q*_(**x**) are the responses of the quadrature filter pair. The ∗ operator corresponds to convolution.

In this context, for calibrated parallel-axis setups, the disparity estimation can be obtained from each oriented filter response (at orientation *θ*_*q*_) by projecting the phase difference along the direction of the (horizontal) epipolar lines. That is, the disparity is defined as the one-dimensional (1D) shift necessary to align, along the direction of the horizontal epipolar lines, the phase values, *ϕ* ^*L*^(**x**) and *ϕ* ^*R*^(**x**), of band-pass filtered versions, *Q* ^*L*^(**x**) and *Q* ^*R*^(**x**), of a stereo image pair *I* ^*R*^(**x**) and *I* ^*L*^ = *I* ^*R*^[*x* + *δ*(**x**)] [84]. That is, in a more formal way,

(4) $$\begin{array}{c} \delta \left(x\right)=\frac{{\left[\phi^{L}\left(x\right)-\phi^{R}\left(x\right)\right]}_{2\pi }}{\omega \left(x\right)}=\frac{{\left[\Delta \phi \left(x\right)\right]}_{2\pi }}{\omega \left(x\right)}, \end{array}$$

where *ω*(**x**) is the average instantaneous frequency of the band-pass signal at point **x** and, under a linear phase model, it can be approximated by *ω*_0_ [65]. However, it is possible to directly obtain the disparity from the main part of phase difference in the complex plane without explicit estimation of the left and right phase. In this way, the wrapping effects on the resulting disparity map are avoided [87]. For that, the following identities are used:

(5) $$\begin{array}{c} {\left[\Delta \phi \left(x\right)\right]}_{2\pi }={\left[\arg\left(Q^{L}Q^{*R}\right)\right]}_{2\pi } \end{array}$$

such that Q* denotes complex conjugate of *Q*. Note that, due to the fact that a bank of oriented Gabor filters is used, the estimation of disparity for each of them should be projected on the horizontal epipolar line. In this way, the detectable disparity range becomes

(6) $$\begin{array}{c} -\frac{\pi }{k_{0}\sin\theta }<d_{x}<\frac{\pi }{k_{0}\sin\theta }, \end{array}$$

where *θ* represents the rotation angle of the Gabor filter and *d*_*x*_ is the horizontal disparity obtained as follows:

(7) $$\begin{array}{c} d_{x}=\frac{\Delta \phi }{k_{0}\sin\theta }. \end{array}$$

Nevertheless, it is necessary to handle horizontal and vertical disparities in order to go towards a more generalized architecture suitable for active stereo vision systems. In this case, disparity is defined as the vector difference in positions of identified corresponding points in the left and right images, each one measured with respect to the fixation point as the origin.

**Figure 3:**
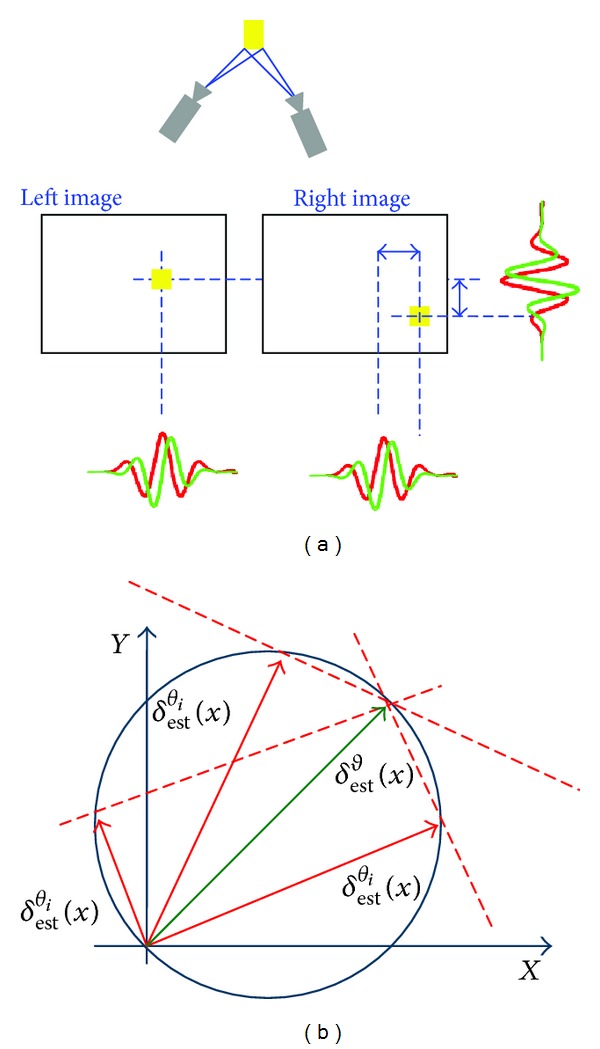
Graphical 2D disparity definition: the difference in the positions of the corresponding points in the stereo image pair (a); the longest vector whose end points lie on the cycle resulting in all the *correct* estimates *δ*_*θ*_^est^ of the disparity component with respect to the orientation *θ* through the origin (b).

In order to estimate the 2D disparity, *δ*(**x**), it is possible to combine the estimates *δ*_*c*,*θ*_ of the bank of filters, oriented by an angle *θ*, by the following formula [77]:

(8) $$\begin{array}{c} \delta^{*}\left(x\right)=\arg\underset{\delta \left(x\right)}{\min}\sum_{\theta }^{}{\left(||\delta_{c,\theta }\left(x\right)||-\delta {\left(x\right)}^{T}\frac{\delta_{c,\theta }\left(x\right)}{||\delta_{c,\theta }\left(x\right)||}\right)}^{2}, \end{array}$$

where *δ*_*c*,*θ*_ denotes the computed disparity along the peak frequency vector of a filter oriented by an angle *θ* and δ*(**x**) is the estimated disparity (see Figure 3).

The equation for handling both horizontal and vertical disparities can be obtained by differentiation of (8), that is

(9) $$\begin{array}{c} \delta^{*}\left(x\right)=\left[\begin{array}{c} \sum_{\theta }^{}d_{x,\theta }\sum_{\theta }^{}d_{y,\theta } \end{array}\right]\left[\begin{array}{cc} \sum_{\theta }^{}\frac{d_{x,\theta }d_{x,\theta }}{d_{x,\theta }^{2}+d_{y,\theta }^{2}} & \sum_{\theta }^{}\frac{d_{x,\theta }d_{y,\theta }}{d_{x,\theta }^{2}+d_{y,\theta }^{2}}\\ \sum_{\theta }^{}\frac{d_{y,\theta }d_{x,\theta }}{d_{x,\theta }^{2}+d_{y,\theta }^{2}} & \sum_{\theta }^{}\frac{d_{y,\theta }d_{y,\theta }}{d_{x,\theta }^{2}+d_{y,\theta }^{2}} \end{array}\right], \end{array}$$

where *d*_*x*,*θ*_ and *d*_*y*,*θ*_ are the projection of *δ*_*c*,*θ*_ along the horizontal and vertical axis, respectively. In this way, multiple disparity estimates are obtained at each location. These estimates can be integrated over the different pyramid levels. For that, a disparity map is first computed at the coarsest level. Then, this disparity estimation is up sampled in order to make it compatible with the next level estimation. For that, an expansion operator and a method to double are used. Although it could be thought that this sample up would result in round off errors and inaccurate disparity estimation, that is not the case. The reason lies in the fact that analyzing images at many scales arises from the nature of images themselves. Actually, scenes in the world contain objects of many sizes, and these objects contain features of many sizes. Moreover, objects can be at various distances from the robot. As a result any analysis method that is applied only at a single scale can miss information at other scales. In addition, image pyramids tend to enhance image features, such as edges, which are important for accuracy in disparity estimation. So, computing a disparity map at the coarsest level allows us to roughly estimate disparity which will be refined at the next level estimation. Therefore, the disparity estimation is more accurate when obtained from different scales. Furthermore, the savings in computation that can be obtained through coarse-fine search can be substantial.

After that sample up, the obtained map is used to reduce the disparity at level *n* + 1, by warping the right filter responses before computing the phase difference

(10) $$\begin{array}{c} \delta^{n}=\frac{{\left[\phi^{L}\left(x\right)-\phi^{R}\left(x^{\prime }\right)\right]}_{2\pi }}{k\left(x\right)}+\left(2\;\text{expand}\left(\delta^{n-1}\right)\right), \end{array}$$

where **x**′ = (*x*+*d*_*x*_^*n*−1^(**x**),*y*+*d*_*y*_^*n*−1^(**x**))^*T*^, with *d*_*x*_^*n*−1^ being the horizontal disparity at level *n* − 1 and *d*_*y*_^*n*−1^ the vertical disparity at level *n* − 1. Consequently, the remaining disparity is guaranteed to lie within the filter range. This procedure is repeated until the finest level is reached.

**Figure 4:**
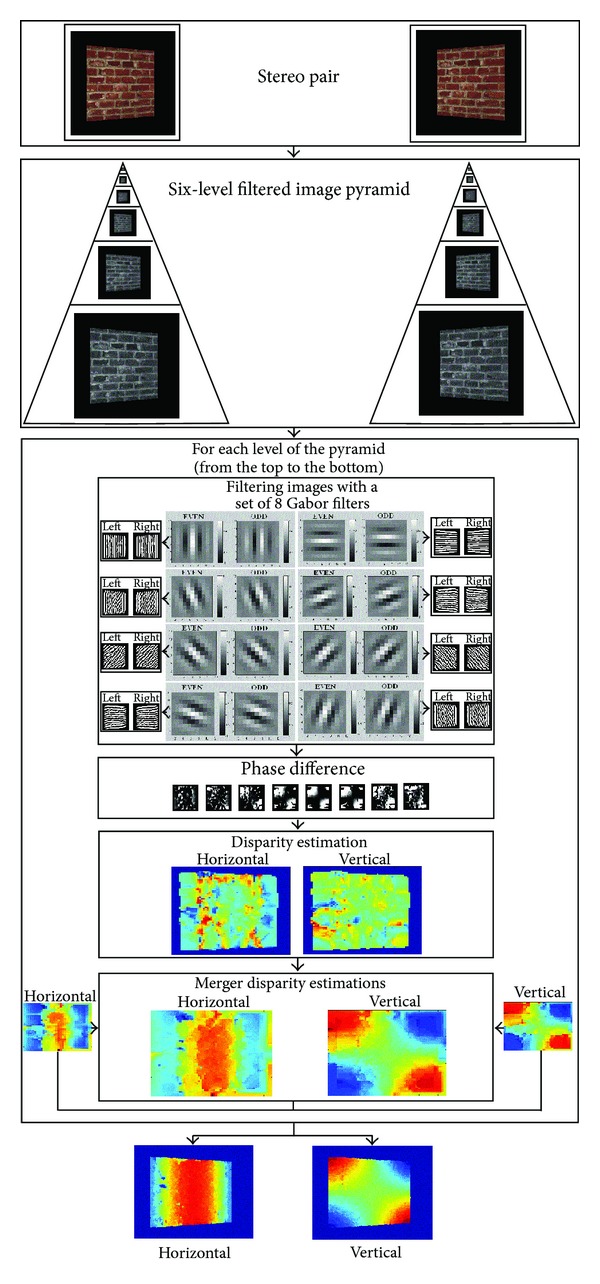
Graphical description of the phase-based binocular disparity estimation (PBBDE) approach: from a captured stereo image pair, an *n*-level image pyramid is built such that a spatial filter is applied to each level for noise reduction. Then, for each level, the images are filtered with a set of eight complex-valued Gabor filters, followed by a phase difference estimation and a following disparity estimation. Finally, the obtained disparity is integrated with the disparity map obtained in the previous level.

Thus, the implemented algorithm, which is depicted in Figure 4, can be summarized as follows.

(1) A stereo image pair is captured.
(2) A six-level image pyramid is built such that a spatial filter is applied to each level for noise reduction. That is, a sequence of copies of an original image, for which both sample density and resolution are decreased in regular steps, is generated. In this way, an efficient scaled convolution can be obtained such as pointed out in [88].
(3) Although, for the sake of clarity, only the processing of the fourth level is depicted in Figure 4, for each level of the generated image pyramid, from the top (the lowest resolution image) to the bottom (the highest resolution image), applies the following.
  (a) Filtering image with a set of eight complex-valued Gabor filters implemented as sums of separable filters as explained in [86] and defined in (4).
  (b) Phase difference estimation between the processed stereo pair. The spatially-localized phase measures, obtained in the previous step through filtering operations, can be expressed as a combination of amplitude (*ρ*(*x*)) and phase (*ϕ*(*x*)) components as follows:
    (11) $$\begin{array}{c} Q\left(x\right)=f_{q}*h\left(x;k_{0}\right)=\rho \left(x\right)e^{i\phi (x)}=C\left(x\right)+iS\left(x\right), \end{array}$$
    where *I* is the processed intensity pattern and *C*(**x**) and *S*(**x**) are the responses of the quadrature filter pair.
  (c) 2D disparity estimation.
  (d) Disparity merging between the current estimation and the one obtained at the previous scale.

Once a disparity estimation is obtained, the next step is to infer the object's depth. For that, two different cases have to be considered.

- Parallel camera axes. In this case, there are only horizontal disparities, but there are no points with zero disparity.
- Convergent camera axes. In this case, there are points with horizontal and/or vertical disparities, but also points characterized by zero disparity. One such point, obviously, is the intersection of the visual axes, that is, the fixation point.

In the simplest case, the camera axes are set parallel to one another and the line which connects the cameras of the stereo camera system, the *baseline* *b*, is at a right angle to them. Consider the image of a point *P* at a distance *z* from the baseline, measured in the direction of the camera axes, and *x*_*l*_ and *x*_*r*_ its position in the left and right images, respectively. So, depth estimation can be obtained by means of the *horizontal disparity* (i.e., *d*_*x*_) in the following way:

(12) $$\begin{array}{c} z=f\frac{b}{x_{r}-x_{l}}=f\frac{b}{d_{x}}. \end{array}$$

Thus, the *d*_*x*_ is inversely proportional to the distance of the point and increases with the focal length *f* and the baseline distance *b*. In camera systems, very long baselines are sometimes used in order to improve the depth resolution.

On the other hand, when a convergent stereo camera system is used, the depth estimation when the fixation and distraction are on the *Y* axis (including sign) and for a given fixate distance *fd* is obtained by the following formula with interocular distance *b*:

(13) $$\begin{array}{c} z=f\frac{4+{\left(b/fd\right)}^{2}}{2\left(\left(b/fd\right)-2\tan\left(\delta /2\right)\right)}\tan\left(\frac{\delta }{2}\right). \end{array}$$

Note that the binocular disparity is expressed in radians in this formula. For that, camera-centred polar coordinates are used. Moreover, depth estimation can be carried out when a fixate distance *fd* exists. The value of *fd* could come from a convergence cue since the convergence angle *α* of the cameras is related to the fixate distance by

(14) $$\begin{array}{c} fd=\frac{b}{2\tan\left(\alpha /2\right)} \end{array}$$

so (after simplification) depth from the visual input of a convergent stereo camera system at any time instant is

(15) $$\begin{array}{c} z=\frac{b}{2}\frac{\sin\left(\delta /2\right)}{\sin\left(\alpha /2\right)\sin\left(\left(\alpha -\delta \right)/2\right)}. \end{array}$$

From a robotic point of view, this binocular depth estimation can be used for motion control, since it provides the required knowledge that allows a perceptual agent to properly interact with its surrounding environment. In particular, we analysed the reaching behaviour of a robotic agent when only an estimated disparity map of its peripersonal space is provided. It is worth noting that, instead of a full metrical 3D reconstruction of the observed scene, a relative representation of the objects that are actively bound on time for the task at hand (in terms of disparity) is used. The equations (13)–(15) are reported to highlight the relationships between disparity and depth, but we use a different approach that uses directly the relative disparity among objects (see Section 3 for details).

With that purpose, an integrated virtual reality environment has been developed. It is an extension of the tool for benchmarking active stereo systems developed in [89] whose aim was to precisely simulate the vergence movements of the two cameras of a stereo vision system. From that starting point, we have developed a tool, based on a C++/OpenGL architecture and on the Coin3D graphic toolkit (http://www.coin3d.org/), that allows us to measure the error in disparity estimation under different situations set by the user (not available in the previous version). So, the tool presented in the following section is aimed at evaluating aspects of a robot acting in an environment. In particular, that tool evaluates the PBBDE accuracy in depth estimation from its 2D estimation without any knowledge of the 3D object and their 2D projections without any knowledge of the 3D object coordinates and their 2D projections. Moreover, those disparity computations are compared with the ground-truth data to estimate the error, which was not done in the previous tool [89]. In addition, some parameters such as, for instance, the inter-ocular distance between the two cameras, the distance between the cameras and the objects, or the fixation points, can be set and/or changed by the user at any time. In typical conditions, the inter-ocular distance between the two cameras was set to 6.5 cm, the distance between the cameras and the objects ranges between 80 and 90 cm, and the fixation points are randomly chosen by using the generated depth map.

## 3. Virtual Robotic Agent Design

**Figure 5:**
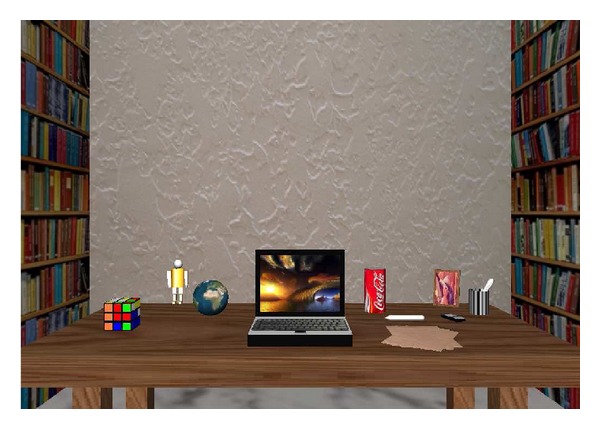
An example of the virtual scene designed for studying the robot behaviour adaptation on reaching tasks from a 3D perception in an unstructured environment.

As an evaluation of our approach's performance, a virtual reality tool has been developed. It allows us to study the adaptation of robots behaviour in reaching tasks triggered by 3D perception in an unstructured environment. In this study, a robotic agent is needed to perform the reaching task. Concretely, it is represented by a robotic arm and a visual system—a pair of stereoscopic vergent cameras—since we are interested in visually controlling the end-effector when the reaching task takes place. The designed virtual scene where the robot acts is divided into two different areas (see Figure 5).

- The agent's peripersonal space, by supposing that the agent is not changing its position in the environment, it was defined as a human peripersonal space, that is, as a hemisphere with a radius of 1.5 metres. As agent-environment interaction was required, the peripersonal space was covered with a set of objects on a 1.5 × 1 × 0.5-metre table. The objects should have different features in order to better test the accuracy of the implemented algorithm. Moreover, in order to create benchmark sequences of appropriate complexity, *realistic* and common-daily objects such as a bin, a portrait, pens, or some pieces of paper are used. It is important to take into account that the different considered features also generate new control issues for the agent when it tries to point at any of those objects.
- A background, which is needed for adding reality to the environment as well as to the disparity map, is composed of walls, roof, and ground, that is, a room.

Thus, the main idea is that, given a scene point, the agent should be capable of setting that point as a fixation point and the target to be reached from the estimated disparity map. In order to avoid the use of a full metrical 3D reconstruction of the observed scene, we use the following strategy both to fixate an object in the scene and to reach it with the arm; given an interest object, the two cameras are moved to bring the interest object in the centre of the foveas, obtaining approximately zero disparity on that object. So, once a target is given for reaching the task, the arm is moved in the proper image position to make the arm's disparity equal to the target's. Therefore, it is important to have dense and reliable disparity maps. In this regard, two different issues have to be considered:

1. the target point is set before changing the fixation point;
2. the target point is set after changing the fixation point.

In the first case, the computed disparity map provides information with respect to the last fixation point. As a consequence, it is possible to directly determine the distance between the current fixation point and the next one from the disparity map since depth measurement is related to the disparity value. Once depth is estimated, displacement along *X*- and *Y*-axis is obtained from the projective camera equations. That is,

- disparity estimation by using the implemented phase-based algorithm,
- depth estimation, *Z*, from the estimated disparity and the parameters of the cameras,
- estimation of the displacement along *X*- and *Y*-axis by using the projective camera equations,
- reference system transformation from head-centred coordinates to arm coordinates.

In the second case, the target point coincides with the fixation point and a different approach follows. Depth information can be inferred from disparity by using (13). So, the disparity map is used to compute depth with the aim of estimating the displacement along *X*- and *Y*-axis. Finally, the 3D coordinates with respect to the arm reference system are obtained from the head-centred frame. In that way, the arm can be properly moved to its next position.

## 4. Experimental Results

In this section, we evaluate the performance of the proposed disparity estimation procedure through several experimental results on both simulated and real environments.

### 4.1. Experimental Results on Simulated Data

Different simulated data are used for assessing the performance of the proposed approach. Firstly, with the aim of evaluating the proposed approach and comparing its accuracy in disparity estimation with other different band-pass representations, some experiments were carried out on image pairs from Middlebury dataset for Stereo Evaluation [42]. Although they only contain horizontal disparities, it provides the disparity ground-truth for all its image sequences, allowing a quantitative comparison between the different approaches.

With the purpose of assessing the accuracy in feature extraction of the proposed method, we analyse and compare different band-pass representations. So, the integer-based measures proposed in the dataset are not used. Instead of them, we compute the mean and standard deviation of the absolute disparity error by comparing the results with the ground-truth. As summarized in Table 1, three classes of filters are used for comparison: Gabor-like kernels, spherical quadrature filters (SQF), and steerable filters (second (s2) and fourth order (s4)). The obtained average and standard deviation of the absolute disparity error, expressed in pixels, highlight that our approach has better results than Gabor filters, which are slightly better than those for the fourth-order steerable filters (s4). The second-order filters (s2), comparable with those obtained by the spherical quadrature filters (SQF), yield results about twice as bad as the fourth-order filters.

**Figure 6:**
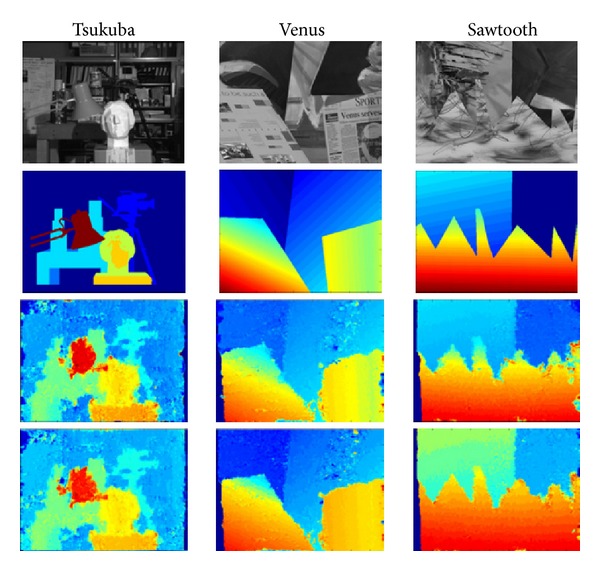
Comparison between horizontal disparity maps on Middlebury images [42] such that the first row corresponds to the left image of the pair; ground-truth disparity is shown in the second row and the computed disparity maps (disparity considered as a one-dimensional shift and a more generalized architecture with both horizontal and vertical disparities) appear in the last two rows, respectively, such that disparity maps are coded from red (positive values of disparity) to blue (negative values).

Figure 6 depicts (from top to bottom) the left images of the stereo-pairs, the ground-truth maps, and the disparity maps obtained with our approach in 1D and 2D by using 6 scales and an energy threshold of 10^−6^.

**Figure 7:**
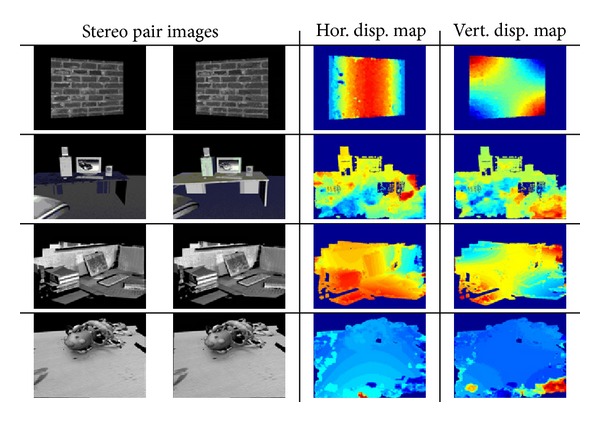
Disparity estimation results obtained on some vergent stereo image pairs generated by a simulator developed in [89] by applying the PBBDE approach. The disparity maps are coded from red (positive values of disparity) to blue (negative values).

Secondly, some experiments were carried out on vergent stereo image pairs generated by the VR simulator developed in [89].  Figure 7 shows some of the obtained results by coding disparity from red (positive values of disparity) to blue (negative values). Again, an image pyramid of 6 scales with an energy threshold of 10^−6^ is used.

In the first example, the two cameras are fixating the centre of a fronto-parallel plane. So, a zero disparity is obtained in the centre of the image in both disparity maps and its value is getting higher as pixels move further from it, that is, towards the borders. Something similar occurs when the fixation point is on the keyboard, the desktop, or the toy, as depicted in Figure 7.

Then, the approach's accuracy is assessed when the integrated virtual reality environment introduced in the previous section is used. The software tool is composed of two different modules:

- a console which the user must initially interact with in order to provide some information about the robotic system configuration (e.g., intercamera distance and head position with respect to the world coordinate system) and,
- an interactive window which consists of two different elements: (i) a main image which is set at the beginning of the experiment and does not change during the whole experiment. It represents the virtual scene seen from a virtual fixed camera and it allows the user to choose, at each time, which is the next fixation point by clicking on the desired point; (ii) a small image, on the top left corner of the window, which represents what is seen by the agent at each time, presented to the user as an anaglyph image.

Each time a user clicks on an object in the virtual scene of the main image, a disparity map is estimated by using the PBBDE approach. Furthermore, a ground-truth disparity is generated for quantitatively measuring the error. Both measurement values, expressed in pixels, are shown in the console, whereas, in the main window, the chosen point becomes the new fixation point for the visual system and it is reached by the agent's arm. It is worth noting that the point chosen as the next fixation point has to be in the binocular field of view because a *full* 3D metrical map of the environment is not built, but only a loose representation of the objects that are actively bound on time.

**Figure 8:**
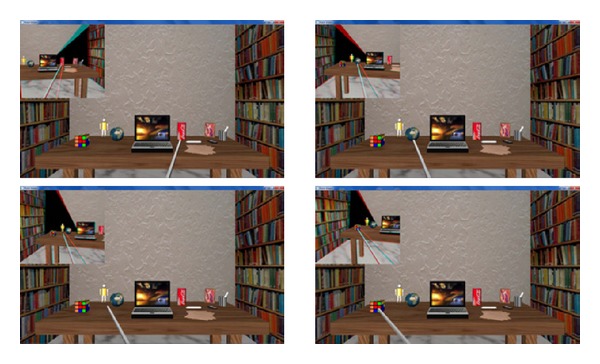
Snapshots of the performance of the virtual developed environment, obtained when the interocular distance between the two stereoscopic vergent cameras is set to 6.5 cm.

**Figure 9:**
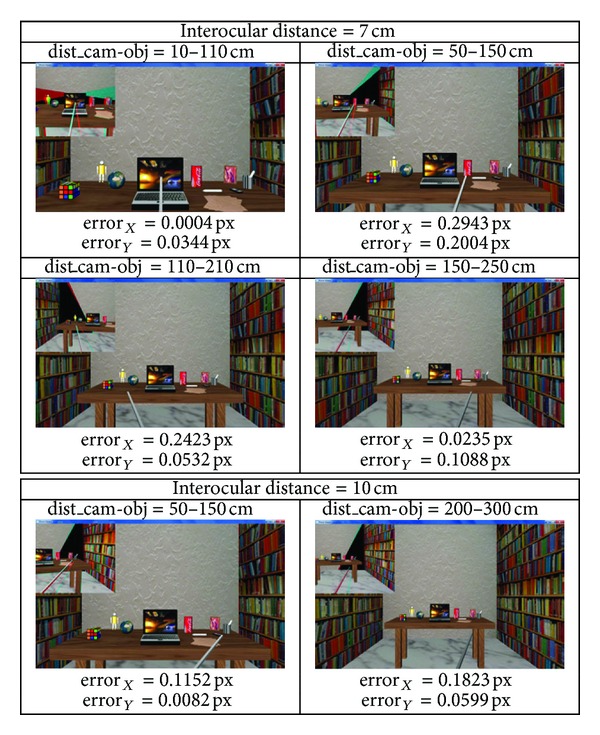
Snapshots of the performances obtained when a different inter-ocular distance and/or the distance between the vision system and the objects ranges is modified.

Figure 8 presents some examples of the designed virtual environment. The simulator aims at mimicking the reaching behaviour of a robotic agent with an active vision system with human-like features acting in the peripersonal space. Therefore, the inter-ocular distance between the two cameras is set to 6.5 cm. The different fixation points have been randomly chosen. Moreover, other experiments with different inter-ocular distance and/or agent distances with respect to the objects were also carried out, quantitatively measuring the error (see Figure 9). These quantitative values are obtained as the difference between the estimated disparity and the ground-truth value in the two considered dimensions, that is, the computed errors for both horizontal (error_*X*_) and vertical (error_*Y*_) disparities expressed in pixels. Note that the disparity errors obtained for the tested points are always under 1.0 pixel, successfully achieving the reaching task in all the cases.

On the other hand, this tool was used to study the parameters affecting the disparity map accuracy. Actually, it is important to properly set those parameters in order to obtain the most accurate disparity maps. With that purpose, experiments on images of different sizes and features were analysed. In all the experiments, ten different levels of the image pyramid were considered, from 1 to 10 scales by steps of 1 scale; and, for each number of levels, ten values were tested for the energy threshold (from 1 to 10^−10^) such that the energy threshold of the next step was obtained as one tenth of the previous one. With the resulting disparity maps, some conclusions could be obtained.

**Figure 10:**
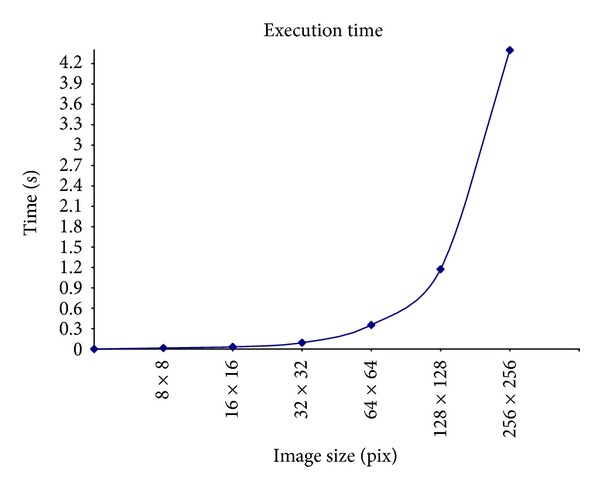
Execution time analysis of the phase-based approach based on the image resolution (in pixels).

- The number of levels of the pyramid depends on the image size. As an image is reduced to one fourth its size in each level, the higher the number of levels is, the less the image resolution is. In fact, it is possible to determine the maximum number of levels to be used from the size of the images.
- The energy threshold depends on how many levels the pyramid has because it is related to the image resolution and, therefore, to the pyramid levels.
- Low energy thresholds do not provide useful information. At least a 10^−3^ energy threshold is needed to obtain any information about disparity.
- Similar results can be obtained with a less number of image pyramid levels if the energy threshold is increased.
- There is a direct relationship between the image resolution and the execution time such that the higher the image resolution is, the slower the performance results. Actually, as shown in Figure 10, it is necessary to work with an image resolution that allows the system to obtain good accuracy without resulting in a high time-consuming application. Note that those results have been obtained by using an Intel(R) Core(TM) Duo CPU P8700 at 2.53 GHz. So, an increase in the computer power will provide better performance with higher resolution images.

### 4.2. Experimental Results on Real Data

The PBBDE approach has also been tested on real environments. The approach's performance has been assessed by means of two laboratory setups. On the one hand, a *STH-DCSG stereo head* was employed [90]. Basically, it is a synchronized digital stereo head camera with two global shutter CMOS imagers, capturing 640 × 480, 24-bit RGB colour images at 30 fps.

Unlike previous evaluation tests, in this case, three different situations have been considered:

1. only horizontal disparity exists,
2. only vertical disparity exists,
3. both horizontal and vertical disparities must be obtained.

**Figure 11:**
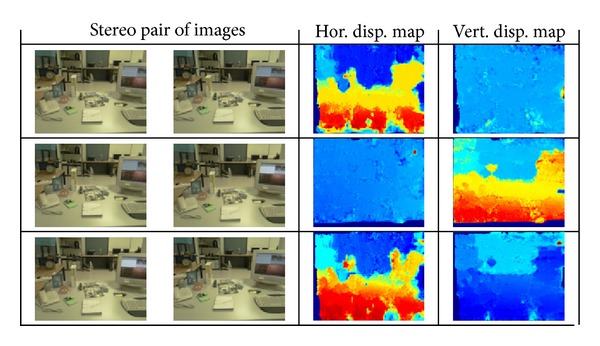
Disparity estimation results obtained on an STH-DCSG stereo head by applying the PBBDE approach when only horizontal disparity appears (first row), only vertical disparity is present (second row), and when both horizontal and vertical disparities appear (last row).

Therefore, depending on the case under study, the obtained disparity maps will be different. So, when only horizontal disparity is present in the images, the vertical disparity map should consist of near zero values; that should happen with the estimated horizontal disparity map when the difference between the two images is just a vertical displacement (case 2). Note that no fixation point has been considered in this case, that is, they have a parallel line-of-sight characteristic. A sample of each considered case together with the estimated disparity maps is shown in Figure 11.

**Figure 12:**
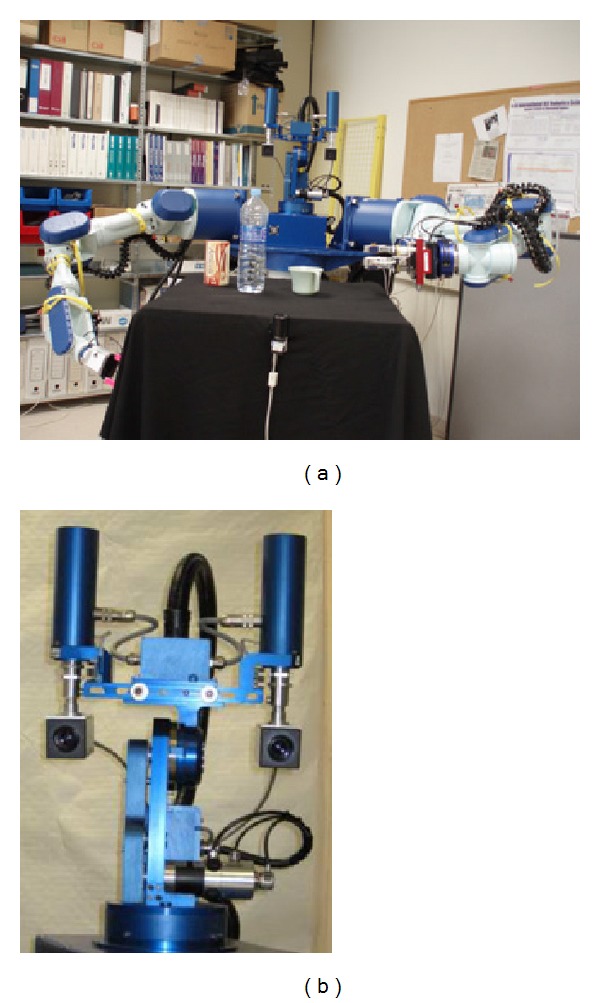
Experimental setup: external view of the used humanoid robot (a) and a detailed view of the pan/tilt/vergence head (b).

Finally, a humanoid robot torso was used (see Figure 12). It is endowed with a pan-tilt-vergent stereo head (*TO40* Head from *Robosoft*) and two multijoint arms (*PA10* arms from *Mitsubishi*) that allow it to perform reaching tasks. The head mounts two cameras with a resolution of 1024 × 768 pixels that can acquire colour images at 30 Hz. The baseline between cameras is 270 mm.

**Figure 13:**
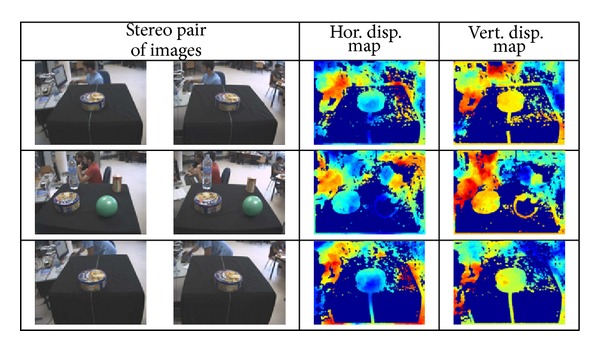
Disparity estimation results obtained by the PBBDE approach on the images acquired by a RoboSoft TO40 setup such that the fixation point is at the cake box and the disparity maps are coded from red (positive values of disparity) to blue (negative values).

**Figure 14:**
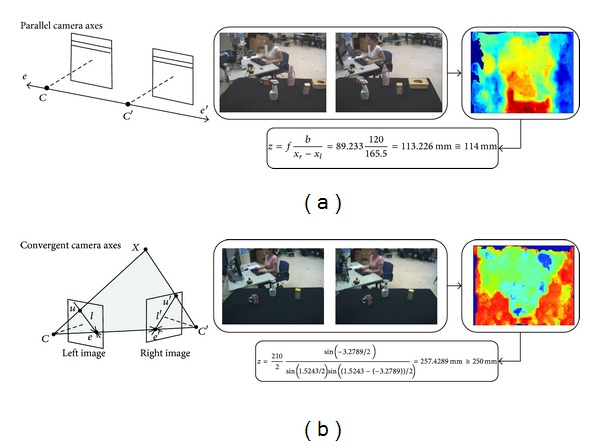
Depth estimation from the disparity maps obtained by the PBBDE approach on the images acquired by a RoboSoft TO40 set-up.

With this experimental setup, a complete performance analysis can be carried out. So, in this case, the depth of the interest object was estimated from the obtained disparity map and compared with the real distance between the robot system and the interest object. Some of the obtained disparity maps are depicted in Figure 13, whereas samples of depth estimation are shown in Figure 14. As can be observed, in both cases, the accuracy of the depth estimation is successful for the task at hand and the obtained error is considerably slow.

## 5. Conclusions and Future Work

In this paper, we have proposed a biological approach that follows the human neural architecture: the motor information is coded in egocentric coordinates obtained from the allocentric representation of the space (in terms of disparity) which, at the same time, is generated from the egocentric representation of the visual information (retinocentric representation). So, as a first step, a binocular depth estimation is carried out. For that, we present PBBDE, a disparity estimation approach that does not require precise calibration information (in terms of the relative orientation of the cameras). Basically, from a set of Gabor filtering, the system provides a disparity map in both *X*- and *Y*-orientations. Thus, instead of a * full* metrical 3D reconstruction of the observed scene, a disparity map of the surrounding space is actively updated on time for the task at hand. That knowledge provides a complete and operative cognition of the environment and can be successfully used for robot motion control.

The performance of the PBBDE approach has been evaluated through several experimental results on both simulated and real environments. Firstly, with the aim of evaluating the accuracy in disparity estimation, Middlebury dataset for Stereo Evaluation [42] has been used. This dataset provides the disparity ground-truth images for all its image sequences. In that way, a quantitative comparison with other band-pass filters could be carried out. As the experiments show, the best results were obtained by the PBBDE approach. In addition, some experiments were performed on vergent stereo image pairs generated by a simulator developed in [89]. With the purpose of testing the PBBDE's performance in the action-perception cycle, an interactive application has been implemented. It evaluates the proposed phase-based approach to estimate disparity maps, such that it allows an agent to estimate depth of target objects to reach them in a reliable way. The obtained results are successful since the maximum error was less than 1.0 pixel, which means that depth estimation will be quite accurate.

On the other hand, the PBBDE's accuracy was also evaluated on real data. For that, a robotic platform mounted with a convergent stereo system was used. In this case, depth estimation from the generated disparity maps was assessed. As the experimental results highlight, the accuracy of the approach was considerably small (less than 1 cm), making the approach suitable for robotic tasks.

Therefore, we have developed a biological strategy which provides a robotic system with basic visuo-motor behaviours, perceptual abilities (depth's perception through disparity estimation), and manipulative abilities (i.e., reaching for a visual target). Therefore, the designed robotic system could robustly perform visuo-motor tasks in complex, natural environments without any *a priori* knowledge.

As a future work, we plan to integrate a visual short-term memory such as that presented recently Brouwer and Knill [91], suggesting that the brain can use that visual short-term memory in a more directed, task-specific manner to help guide reaching movements. So, rather than relying on visual information alone when it is available and reliable, humans appear to use both sources of information to the limits of their reliability. In addition, we would like to investigate the memory capacity and reference frames used for storing object information for the use in action in a robotic platform.

## Figures and Tables

**Table 1:** Quantitative comparison (average and standard deviation of the absolute errors in pixels) in disparity estimation between different band-pass representations on Middlebury images [42].

Algorithm	Tsukuba		Venus		Sawtooth	
	Avg.	Std.	Avg.	Std.	Avg.	Std.
Gabor	0.32	0.61	0.25	0.77	0.41	1.26
s4	0.36	0.68	0.40	1.30	0.5	1.86
s2	0.47	0.79	0.98	2.44	1.12	2.50
SQF	0.46	0.85	0.95	2.40	0.93	2.20
PBBDE						
1D shift	0.28	0.64	0.99	0.74	0.74	1.10
2D shift	0.12	0.67	0.04	0.74	0.57	1.08
